# Mechanistic insights and the clinical prospects of targeted therapies for glioblastoma: a comprehensive review

**DOI:** 10.1186/s40164-024-00512-8

**Published:** 2024-04-13

**Authors:** Yating Shen, Dexter Kai Hao Thng, Andrea Li Ann Wong, Tan Boon Toh

**Affiliations:** 1 The N.1 Institute for Health (N.1), National University of Singapore, Singapore, Singapore; 2 Cancer Science Institute of Singapore, National University of Singapore, Singapore, Singapore; 3 Department of Haematology-Oncology, National University Hospital, Singapore, Singapore; 4 The Institute for Digital Medicine (WisDM), National University of Singapore, Singapore, Singapore

**Keywords:** Glioblastoma, Targeted therapy, Combinatorial therapy, Clinical assessment

## Abstract

Glioblastoma (GBM) is a fatal brain tumour that is traditionally diagnosed based on histological features. Recent molecular profiling studies have reshaped the World Health Organization approach in the classification of central nervous system tumours to include more pathogenetic hallmarks. These studies have revealed that multiple oncogenic pathways are dysregulated, which contributes to the aggressiveness and resistance of GBM. Such findings have shed light on the molecular vulnerability of GBM and have shifted the disease management paradigm from chemotherapy to targeted therapies. Targeted drugs have been developed to inhibit oncogenic targets in GBM, including receptors involved in the angiogenic axis, the signal transducer and activator of transcription 3 (STAT3), the PI3K/AKT/mTOR signalling pathway, the ubiquitination-proteasome pathway, as well as IDH1/2 pathway. While certain targeted drugs showed promising results in vivo, the translatability of such preclinical achievements in GBM remains a barrier. We also discuss the recent developments and clinical assessments of targeted drugs, as well as the prospects of cell-based therapies and combinatorial therapy as novel ways to target GBM. Targeted treatments have demonstrated preclinical efficacy over chemotherapy as an alternative or adjuvant to the current standard of care for GBM, but their clinical efficacy remains hindered by challenges such as blood-brain barrier penetrance of the drugs. The development of combinatorial targeted therapies is expected to improve therapeutic efficacy and overcome drug resistance.

## Introduction

Glioblastoma (GBM) is the most common and lethal malignancy that makes up about 75% of all adult gliomas [1, 2]. The prognosis for GBM is poor, with a median survival of 12–15 months and a five-year survival rate of 6.9% [3]. The Stupp protocol, which comprises of concomitant radiation and chemotherapy, temozolomide (TMZ) to destroy cancer cells, is used as the first line of therapy for GBM. The Stupp protocol has been the standard of treatment since 2005 after reported to have improved two-year survival from 10.4 to 26.5% as compared to radiation alone [4]. However, long-term treatment success is limited as more than 90% of GBM patients still develop resistance and suffer relapse [5], highlighting the limitations of this standard regimen. This has prompted studies aimed at understanding mechanisms that drive rapid growth and proliferation of GBM, as well as modulation of the tumour microenvironment to support tumour growth, invasion, and resistance. While GBM remains highly lethal and difficult to treat, understanding this mechanism will pave the way for the development of better targeted therapeutics.

Traditionally, gliomas are classified based on histological features. Advancements in molecular profiling have allowed for a more accurate classification based on genetic and epigenetic characteristics. This refinement in patient stratification paves the way for better disease management. A study by Wen and Kesari described pathogenetic hallmarks of primary GBMs including amplified epidermal growth factor receptor (*EGFR*), loss of heterogeneity of chromosomal 10q, and deletion of phosphatase and tensin homologue on chromosome 10 (*PTEN*) and p16 [6]. Following the identification of these key genetic aberrations in GBM, Eckel-Passow et al. investigated the significance and association to survival of three markers for defining molecular groups in gliomas, namely chromosome arms 1p and 19q, *IDH* mutations, and *TERT* promoter mutations. *TERT* promoter mutations are the most common (74%) in GBM patients, distinguishing GBM from low grade gliomas. Patients with glioma who solely have *TERT* mutations have poorer prognosis than those who also have *IDH* mutations and/or 1p/19q codeletion [7].

Building on the basis of above-mentioned landmark findings, the current version of the WHO Classification of Tumours of the Central Nervous System 5th edition (WHO CNS 5) classifies GBM as diffused astrocytic glioma, characterised by *isocitrate dehydrogenase* (*IDH*)-WT with the presence of other key genetic alterations, such as mutations in *TERT* promoter, amplification of *EGFR*, and concurrent gain of chromosome 7 and loss of chromosome 10 [8]. Phillips et al. further categorized GBM into three subgroups based on differential expression of particular markers, namely the proneural, proliferative, and mesenchymal subtypes [9]. Following this landmark study, Verhaak et al. performed a more in-depth analysis of genomic data from the TCGA-GBM cohort and classified GBM into four subtypes - Proneural, Neural, Classical and Mesenchymal, of which the neural subtype was later confirmed to be contaminated with normal neural cells and thus removed from the classification [10, 11]. Since then, there has been a surge in interest on the utilization of targeted drugs against genomic aberrations identified in respective subtypes, including *EGFR* amplification in the classical subtype, *NF-kB* hyperactivation in the mesenchymal subtype and *PDGFR* mutation in the proneural subtype.

In this review, we discuss the therapeutic potential and clinical assessment of drugs targeting common oncogenic pathways in GBM. Additionally, we also describe the recent advancement in cell-based therapies against GBM. Finally, we explore the prospects of combinatorial therapies in improving the clinical outcomes for GBM patients.

## Targeted therapy

**Fig. 1:**
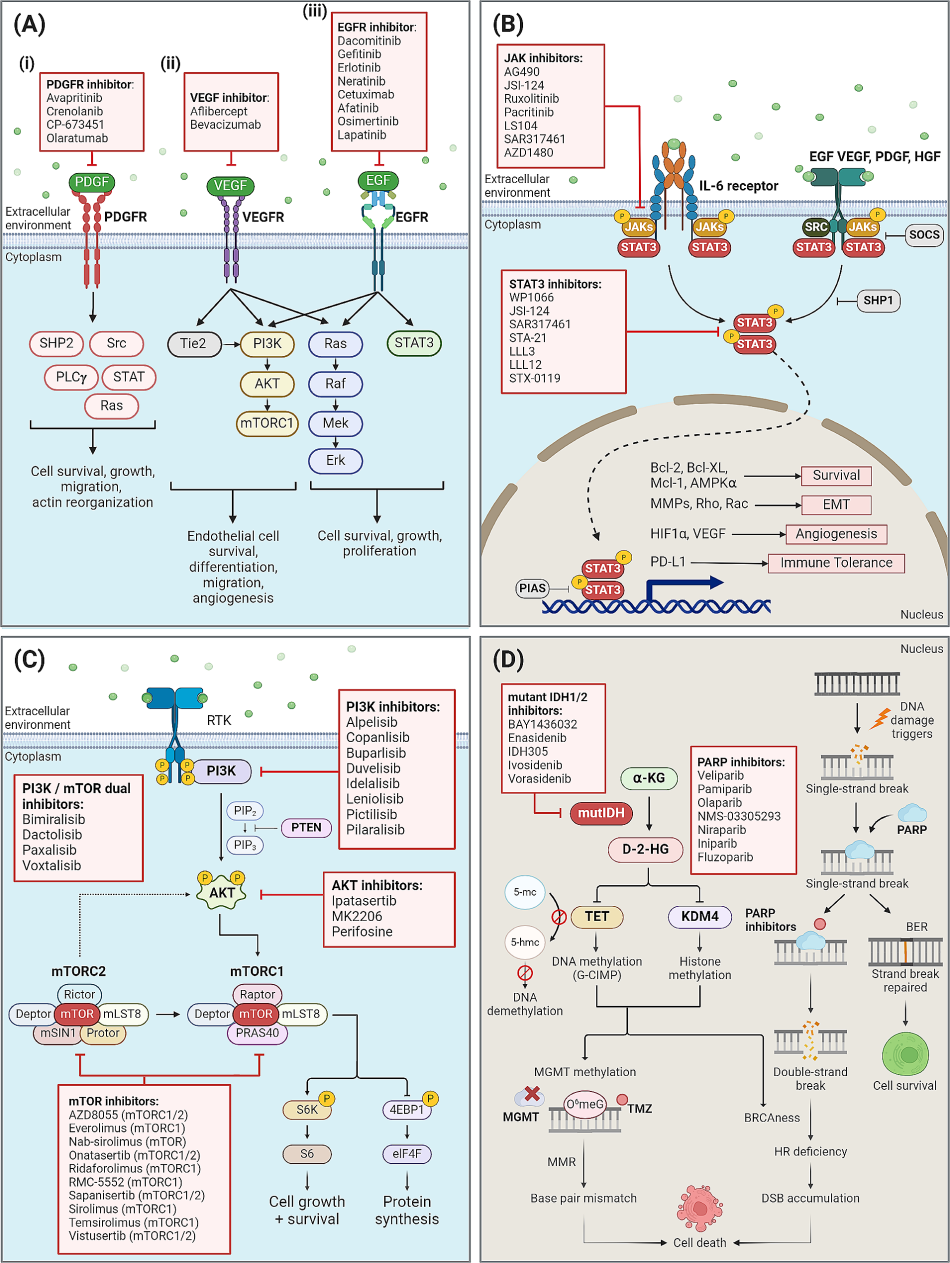
Schematic representation of key regulatory pathways in implicated in gliomagenesis, and corresponding inhibitors that have been investigated in GBM. **(A)** PDGFR, VEGFR, EGFR as oncogenic receptors implicated in the angiogenesis axis. **(i)** Overexpression of PDGF and PDGFR in GBM cells collectively lead to increased tumour cell survival, growth, and migratory capability. **(ii)** Abnormal activation of the VEGFR pathway is responsible for the development of the characteristic leaky neovasculature found in GBM through promoting endothelial cell survival, differentiation, and migration, as well as encouraging microvascular formation. **(iii)** EGFR is commonly overexpressed in GBM and is implicated in improving tumour cell survival, growth, and proliferation through multiple pathways. **(B)** STAT3 can be activated by multiple receptors including IL-6 receptor, EGF, PDGF and HGF via the JAK/STAT signalling pathway. STAT3 activation is modulated by both upstream and downstream regulators. However, STAT3 is often upregulated in GBM cells, thus resulting in overexpression of STAT3 target genes, leading to gliomagenesis. **(C)** Overexpression of RTK and deficiency of the negative regulator PTEN in GBM cells collectively lead to the hyperactivation of AKT, which in turn mediates the activation of downstream kinases that regulate cell proliferation and protein synthesis. **(D)** Healthy cells depend on PARP to repair single-strand breaks. Inhibition of PARP-dependent DNA repair results in accumulation of double strand-breaks, which induce the activation of homologous recombination as the compensatory pathway to repair DNA damage. Oncometabolite D-2-HG blocks the activation of this compensatory pathway through induction of BRCAness, hence offering PARP as a synthetic lethality target in *IDH*-mutant GBM cells. Inhibition of all these targets (in red boxes) therefore serves as potential therapeutic approaches in treating GBM.

Targeted therapy aims at treating cancers by using drugs or monoclonal antibodies that target specific genes or proteins that are critical for the growth or survival of the cancers. This contrasts with conventional cytotoxic chemotherapy that negatively affects both normal and cancer cells. Targeted therapies minimize the off-target toxicities in normal cells and have gained traction over the past decades. A notable example of clinically-approved targeted therapy is bevacizumab, a vascular endothelial growth factor receptors (VEGFR) inhibitor, approved by the U.S. Food and Drug Administration (FDA) for treatment of progressive recurrent GBM [12]. Following, we examine various therapeutic approaches targeting the angiogenic axis, the JAK/STAT pathway, IDH1/2, and the protein clearance system in GBM (Fig. 1).

### Targeting angiogenic axis in GBM

GBM tumours frequently exhibit extensive abnormal vasculature necessary for rapid tumorigenesis. Studies have therefore investigated the role of growth factor receptors involved in angiogenesis, such as the platelet-derived growth factor receptor (PDGFR), vascular endothelial growth factor receptor (VEGFR), and epidermal growth factor receptor (EGFR), contributing to the development of GBM as well as the therapeutic potential of these targets (Fig. 1A).

PDGF signalling is conventionally involved in the growth and differentiation of cells of mesenchymal origin [13]. In embryonic development, PDGFs are known to play a significant role in encouraging development of the central nervous system by supporting oligodendrocyte precursor growth [14]. PDGFR activation has numerous downstream effects including but not limited to activation of Src, SHP-2 tyrosine phosphatase, Phospholipase Cγ, Ras, and various STAT proteins [15] (Fig. 1A). PDGFR activation positively regulates cell proliferation and survival, as well as actin reorganization and cellular migration. Importantly, increased activity of the PDGFR signalling pathway has been observed in high grade gliomas. Glioma cell lines and primary GBM tissues have demonstrated overexpression of both PDGFs and PDGFRs [16]. This is particularly prevalent in the proneural subtype of GBM, which exhibits a high rate of focal *PDGFRA* amplification (35%), making *PDGFRA* amplification a specific hallmark of the proneural GBM [11]. Furthermore, expression of PDGFRβ has been observed in the endothelial cells of GBM but not in the vessels of normal human brain, demonstrating its potential as a GBM-specific targeted therapy [17].

To date, a few anti-PDGFR pharmacological agents have been developed and demonstrated efficacy in mitigating GBM tumorigenesis. Anti-PDGFRα antibody, olaratumab, was found to have promising results in GBM xenografts, significantly inhibiting tumour growth in vivo with a concomitant reduction in PDGFR phosphorylation [18, 19]. Alternatively, small molecule inhibitor against both PDGFR⍺ and PDGFRβ, CP-673,451, inhibits tumour growth by inducing terminal differentiation of GBM cells into neural-like cells [20, 21]. Another inhibitor of PDGFR⍺ and PDGFRβ is crenolanib (CP-868,596), which has demonstrated brain penetration as well as in vivo inhibition of PDGFR phosphorylation in murine models and in high grade glioma patients [22, 23]. Avapritinib and ripretinib are PDGFR inhibitors developed and approved for the treatment of PDGFR⍺ mutant gastrointestinal stromal tumour (GIST) [20, 21]. Unlike other PDGFR inhibitors, avapritinib and ripretinib are more potent as they selectively bind to the activation loop of PDGFR⍺ [24]. Among these two inhibitors, avapritinib is a more promising inhibitor for GBM for its high CNS penetrance as compared to ripretinib [22, 23]. Currently, only olaratumab has successfully progressed to clinical trials for GBM (Table 1). Despite its therapeutic potential in pre-clinical models of GBM, olaratumab showed minimal clinical efficacy in GBM patients, with a median overall survival (OS) of 34.3 weeks compared to the VEGFR inhibitor, ramucirumab (49.5 weeks) [25]. Though no trial has been conducted on the use of crenolanib and avapritinib in GBM patients specifically, trials conducted on high-grade glioma patients suggested that crenolanib has limited additional benefits [26], while avapritinib demonstrated more promising response [22, 27]. More clinical data are therefore needed for the aforementioned PDGFR inhibitors in order to assess their efficacy as prospective inhibitors for GBM.

**Table 1:** Clinical studies of recent targeted therapies for GBM, as single agent or in combination with standard of care. (Data from http://clinicaltrials.gov was searched until Nov 2023)

Target	Drug	Adjuvant Therapy	Phase	Patient Group	NCT number	Safety profile^1^	RANO criteria^2^	PFS^2^	OS^2^
PDGFR	Olaratumab	–	2	rGBM	NCT00895180 (Completed)	-	Yes	PFS-6: 7.5%	Median: 34.3 weeks
VEGF	Bevacizumab	TMZ, RT	3	ndGBM	NCT05271240 (Recruiting)	-	Yes	Ongoing	Ongoing
	Bevacizumab	TMZ, RT	3	rGBM	NCT02761070 (Active, not recruiting)	-	No	Ongoing	Ongoing
	Bevacizumab	TMZ	2	GBM	NCT02898012 (Completed)	-	Yes	Median: 15.3 weeks	Median: 23.9 weeks
	Vatalanib	TMZ, RT	1/2	ndGBM	NCT00128700 (Completed)	Well tolerated	Yes	Median: 7.2 months	Median: 16.2 months-
EGFR	Erlotinib	–	1	rGBM	NCT01257594 (Completed)	Unreported	-	-	-
	Afatinib	TMZ, RT	1	ndGBM	NCT00977431 (Completed)	MTD: 30 mg with daily TMZ/RT and 40 mg with RT alone	-	-	-
	Afatinib	TMZ	2	rGBM	NCT00727506 (Completed)	-	Yes	Median: 0.99 months, 1.53 months with TMZ PSF-6: 3.0% and 10.3% with TMZ	Unreported
	Afatinib	–	2	GBM	NCT00875433 (Completed)	-	No	Median: 10.6 weeks	Median: 39.6 weeks
	Dacomitinib	–	2	rGBM	NCT01112527 (Completed)	-	No	Unreported	Unreported
	Dacomitinib	–	2	rGBM	NCT01520870 (Completed)	-	Yes	Median: 2.7 months PFS-6: 10.6%	Median: 7.4 months
	Neratinib	–	2	GBM	NCT02977780 (Recruiting)	-	Yes	Median: 4.7 months	Median: 14.9 months
	Cetuximab	RT	2	rGBM	NCT02800486 (Recruiting)	-	No	Ongoing	Ongoing
	Cetuximab	–	1/2	ndGBM	NCT02861898 (Recruiting)		Yes	Ongoing	Ongoing
	Cetuximab	RT, TMZ	2	ndGBM	NCT01044225 (Terminated, non-drug related)	-	No	Unreported	Unreported
STAT3	WP1066	–	1	rGBM	NCT01904123 (Completed)	MFD: 8 mg/kg	-	-	-
	WP1066	RT	2	ndGBM	NCT05879250 (Not yet recruiting)	-	Yes	Ongoing	Ongoing
PI3K	Buparlisib	RT, TMZ	1	rGBM	NCT01473901 (Completed)	Not tolerable	-	-	-
	Buparlisib	–	2	rGBM	NCT01339052 (Completed)	-	Yes	Median: 1.7 months PFS-6: 8%	Median: 9.8 months
	Sonolisib	–	2	GBM	NCT01259869 (Completed)	-	Yes	Median: PFS-6: 17%	Unreported
mTOR	Everolimus	TMZ, RT	1/2	ndGBM	NCT01062399 (Completed)	Combining with TMZ increased toxicity	Yes	Median: 8.2 months	Median: 16.5 months
	Sirolimus	–	1/2	GBM	NCT00047073 (Completed)	Tolerable	No	Unreported	Unreported
	Metformin	TMZ	2	ndGBM	NCT04945148 (Not yet recruiting)	-	Yes	Ongoing	Ongoing
	Vistusertib	–	1	rGBM	NCT02619864 (Completed)	Tolerable	-	-	-
	AZD8055	–	1	rGBM	NCT01316809 (Completed)	Unreported	-	-	-
	Onatasertib	–	1/2	Advanced solid tumours	NCT01177397 (Completed)	Tolerable	Yes	PFS-6: 0%	Unreported
	RMC-5552	–	1/1b	rGBM	NCT05557292 (Active, not recruiting)	Ongoing	-	-	-
	Sapanisertib	–	1	rGBM	NCT02133183 (Active, not recruiting)	Unreported	-	-	-
PI3K/mTOR	Paxalisib	RT, TMZ	2	ndGBM	NCT03522298 (Active, not recruiting)	-	Yes	Median: 8.4 months	Median: 15.7%
	Voxtalisib	RT, TMZ	1	GBM	NCT00704080 (Completed)	MTD: 90 mg q.d. and 40 mg b.i.d. with TMZ	-	-	-
Proteasome	Bortezomib	TMZ	2	rGBM	NCT03643549 (Recruiting)	-	Yes	Ongoing	Median: 12.7 months for unmethylated, 21.7 months for methylated
	Bortezomib	RT, TMZ	2	ndGBM	NCT00998010 (Completed)	-	Yes	Median: 6.2 months	Median: 19.1 months
	Marizomib	RT, TMZ	1/2	ndGBM	NCT02903069 (Completed)	Tolerable	Yes	Unreported	Median: 14.8 months
	Marizomib	RT, TMZ	3	ndGBM	NCT03345095 (Completed)	-	Yes	Median: 6.34 months	Median: 16.13 months
	Disulfiram (+ Copper gluconate)	RT, TMZ	1	GBM	NCT01907165 (Completed)	Safe but can cause reversible neurological toxicities	-	-	-
	Disulfiram (+ Copper gluconate)	TMZ	2	rGBM	NCT03034135 (Completed)	-	Yes	Median: 1.7 months	Median: 7.1 months
	Disulfiram (+ Copper gluconate)	TMZ	2	Unmethylated GBM	NCT03363659 (Terminated, non-drug related)	-	No	PFS-6: 8.3%	69.2% after 1 year, 15.4% after 2 years
	Disulfiram (+ Copper gluconate)	RT, TMZ	1/2	ndGBM	NCT02715609 (Active, not recruiting)	MTD: 375 mg/kg	Yes	Ongoing	Ongoing
PARP	Olaparib	TMZ	1	rGBM	NCT01390571 (Completed)	Tolerable	-	-	-
	Olaparib/ Pamiparib	TMZ, RT	1/2	rGBM	NCT04614909 (Recruiting)	Ongoing	No	Ongoing	Ongoing
	Olaparib	TMZ, RT	1/2	GBM	NCT03212742 (Recruiting)	Tolerable	No	Ongoing	Ongoing
	Olaparib	–	Early 1	rGBM	NCT05432518 (Recruiting)	Ongoing	-	-	-
	Olaparib	–	2	rGBM	NCT03212274 (Active, not recruiting)	-	Yes	Ongoing	Ongoing
	Niraparib	–	2	rGBM	NCT05297864 (Active, not recruiting)	-	Yes	Ongoing	Ongoing
	Niraparib	RT	2	rGBM	NCT04221503 (Active, not recruiting)	-	Yes	Ongoing	Ongoing
	Niraparib	RT	2	rGBM	NCT04715620 (Unknown status)	-	No	Median: 157 days PFS-6: 36%	Median: Ongoing OS-6: 82.61%
	Niraparib	RT	Early 1	ndGBM	NCT05076513 (Recruiting)	Tolerable	-	-	-
	Niraparib	TMZ	Early 1	rGBM	NCT01294735 (Completed)	MTD: 40 mg niraparib + 150mg/m^2^ TMZ	-	-	-
	BSI-201	TMZ	1/2	ndGBM	NCT00687765 (Completed)	Unreported	No	Unreported	Unreported
	BGB-290	TMZ	1/2	rGBM	NCT03914742 (Completed)	Unreported	Yes	Unreported	Unreported
	BGB-290	TMZ	1	GBM	NCT03749187 (Recruiting)	Ongoing	-	-	-
	Veliparib	TMZ, RT	2	ndGBM	NCT03581292 (Active, not recruiting)	-	No	Ongoing	Ongoing
	Veliparib	TMZ, RT	1/2	ndGBM	NCT01514201 (Completed)	Tolerable	No	PFS-3: 2.9%	OS-3: 5.3%
	Fluzoparil	TMZ	2	rGBM	NCT04552977 (Unknown status)	-	No	Unreported	Unreported
	NMS-03305293	TMZ	1/2	rGBM	NCT04910022 (Recruiting)	Ongoing	Yes	Ongoing	Ongoing

GBM, glioblastoma; rGBM, recurrent glioblastoma; ndGBM, newly diagnosed glioblastoma; RT, radiotherapy; TMZ, temozolomide; MTD, maximum tolerated dose; RANO, Response assessment in neuro-oncology criteria; PFS-3/6, 3/6 months progression free survival; OS-3/6, 3/6 year overall survival

^1^Only in Phase 1 trials; ^2^Only in Phase 2 and Phase 3 trials

VEGF is a key signalling protein that mediates angiogenesis in response to hypoxia. Activation of VEGF-receptor 2 (VEGFR2) upon binding of VEGF ligand induces the activation of oncogenic PI3K/AKT, MAPK and angiopoietin-Tie pathways [28, 29] (Fig. 1A). In high grade gliomas, VEGFs are often highly expressed, leading to disorganization of tumour vasculature and leaky blood-brain barrier (BBB) [28, 29]. Bevacizumab (BEV), a recombinant humanized monoclonal antibody against all isoforms of VEGFs, is the most frequently investigated VEGF inhibitor, with more than 100 clinical trials evaluating its efficacy singly or in combination for both primary and recurrent GBM patients. After promising results in two Phase 2 trials [30], the FDA granted BEV expedited approval in 2009 for use as a single drug in the treatment of recurrent GBM [31]. In the BRAIN trial, which investigated BEV as a single agent and in combination with irinotecan, the reported six-month progression free survival (PFS6) was 42% in single-agent BEV and 50% in BEV plus irinotecan, with OS of 37% and 35% in respective arms [30]. The results indicate that, although both regimens exceeded benchmark records, combinatorial treatment was comparable with single-agent treatment [30]. Additionally, trials of BEV in combination with other treatment modalities in primary GBM also showed disappointing results [32–34]. Importantly, subsequent studies pointed out that the favourable progression free survival (PFS) observed after BEV treatment may be attributed to pseudoresponse, which gave rise to the brief improvement in radiologic response rather than true tumour shrinkage [35]. Hence, the potential anti-tumour effect of BEV in GBM warrants further validation.

Subsequently, more studies investigated the efficacy of BEV in combination with other chemotherapies (Table 2). Notably, there has been an surge in clinical trials investigating the value of BEV in combination with immunomodulatory agents, such as PD-1 antagonists. Several studies have demonstrated that anti-angiogenic agents can reprogramme the immunosuppressive tumour microenvironment to an immunosupportive one, potentially sensitizing patients to immunotherapies in combination with antiangiogenic therapies [36]. In a recent case report, BEV in combination with pembrolizumab improved the overall survival (OS) of a GBM patient with extracranial metastases to 27 months, higher than the median OS of 11–17 months [37]. However, the response to these combinations in larger clinical trials exhibited only a modest improvement in PFS6 between cohorts who received BEV monotherapies (6.7%) and BEV in combination with pembrolizumab (26.0%) [38]. This suggests that clinical response to these combination therapies is still largely varied and warrants the identification of predictive biomarkers for patient stratification towards BEV with immunomodulatory agents. Subsequent trials are therefore investigating the potential of BEV with various anti-PD-1 agents while simultaneously altering BEV dosing schedule and performing biomarker correlative studies (Table 2).

**Table 2:** Clinical studies of recent targeted combinatorial therapies in GBM. (Data from http://clinicaltrials.gov was searched until Nov 2023)

Targets	Drug combination	Phase	Patient Group	NCT number	Safety profile^1^	RANO criteria^2^	PFS^2^	OS^2^
VEGF, PD-1	Bevacizumab + Nivolumab	2	GBM	NCT03452579 (Active, not recruiting)	-	Yes	Unreported	OS-1: 46.2% for standard bevacizumab, 37.7% for low dose bevacizumab
VEGF, PD-1	Bevacizumab + Nivolumab	2	GBM	NCT03743662 (Active, not recruiting)	-	Yes	Ongoing	Ongoing
VEGF, PD-1	Bevacizumab + Camrelizumab	2	rGBM	NCT04952571 (Suspended, non-drug related)	-	No	Suspended	Suspended
VEGF, PD-1	Bevacizumab + Pembrolizumab	2	rGBM	NCT03661723 (Active, not recruited)	-	Yes	Median: 4 months PFS-6: 10.6%	Median: 8.6 months OS-6: 56.7% OS-12: 16.6%
VEGF, PD-1	Bevacizumab + Tislelizumab	2	rGBM	NCT05540275 (Not yet recruiting)	-	Yes	Ongoing	Ongoing
VEGF, PD-1	Bevacizumab + Sintilimab	2	rGBM	NCT05502991 (Not yet recruiting)	-	Yes	Ongoing	Ongoing
VEGF, PD-1	Bevacizumab + Durvalumab	2	rGBM	NCT02336165 (Completed)	-	Yes	Median: 16 weeks PFS-6: 15.2%	Median: 19.3 weeks OS-6: 36.4%
VEGF, T-cell	Bevacizumab + GX-17	2	rGBM	NCT05191784 (Active, not recruiting)	-	Yes	Ongoing	Ongoing
VEGF, endoglin	Bevacizumab + TRC105	2	GBM	NCT01564914 (Completed)	-	Yes	Median: 1.81 months	Median: 5.75 months
VEGF, endoglin	Bevacizumab + TRC105	1/2	rGBM	NCT01648348 (Completed)	Tolerable, MTD: 10 mg/kg bevacizumab + 10 mg/kg TRC105	Yes	Median: 2.9 months PFS-6: 25%	Median: 9.7%
VEGF, PI3K	Bevacizumab + BKM120	1/2	rGBM	NCT01349660 (Completed)	Tolerable at 60 mg/day BKM120	Yes	Median: 2.8 months for prior bevacizumab, 5.3 months for bevacizumab naïve	Median: 6.6 months for prior bevacizumab, 10.8 months for bevacizumab naïve
VEGF, mTOR	Bevacizumab + Everolimus	2	GBM	NCT00805961 (Completed)	-	Yes	Median: 11.3 months	Median: 13.9 months
VEGF, mTOR	Bevacizumab + nab-sirolimus	2	ndGBM	NCT03463265 (Completed)	-	Yes	Median: 3.1 months PFS-6: 37.5% PFS-12: 12.5%	Median: 6.8 months OS-12: 25%
VEGF, mTOR	Bevacizumab + Sapanisertib	1	rGBM	NCT02142803 (Active, not recruiting)	MTD/RP2D: 5 mg/day	-	-	-
VEGF, angiopoietin1/2	Bevacizumab + Trebananib	1/2	rGBM	NCT01290263 (Completed)	Tolerable	Yes	Median: 285 days PFS-6: 24.3%	Median: 108 days
VEGF, angiopoietin1/2	Bevacizumab + Trebananib	2	rGBM	NCT01609790 (Completed)	-	Yes	Median: 4.2 months PFS-6: 22.6%	Median: 7.5 months
VEGF, proteasome	Bevacizumab + Marizomib	1/2	GBM	NCT02330562 (Completed)	Tolerable, RP2D: MRZ 0.8 mg/m^2^	Yes	Median: 3.9 months	Median: 10.4 months
VEGF, CDK4/6	Bevacizumab + Abemaciclib	1	rGBM	NCT04074785 (Active, not recruiting)	Ongoing	-	-	-
VEGF, multi-kinases	Bevacizumab + Ponatinib	2	rGBM	NCT02478164 (Completed)	-	Yes	Median: 28 days PFS-3: 0%	Median: 98 days
VEGF, FASN	Bevacizumab + ASC40	3	rGBM	NCT05118776 (Recruiting)	-	Yes	Ongoing	Ongoing
VEGF, topoisomerase I	Bevacizumab + Irinotecan	1	rGBM	NCT05201326 (Recruiting)	Ongoing	-	-	-
VEGF, angiogenesis	Bevacizumab + VB-111	3	rGBM	NCT02511405 (Completed)	-	Yes	Median: 3.4 months	Median: 6.8 months
EGFR, glucose	Osimertinib + Fludeoxyglucose F-18	2	rGBM	NCT03732352 (Active, not recruiting)	-	Yes	Ongoing	Ongoing
EGFR, Osmolality	Cetuximab + Mannitol	1/2	GBM	NCT02861898 (Recruiting)	Ongoing	Yes	Ongoing	Ongoing
EGFR, multi-kinases	Erlotinib + Sorafenib	2	rGBM	NCT00445588 (Completed)	-	No	PFS-6: 14%	Median: 5.7 months
PI3K, c-MET	Buparlisib + INC280	1/2	rGBM	NCT01870726 (Terminated, no clear activity)	RP2D not declared due to drug-drug interactions	Yes	Combination arms not initiated	Combination arms not initiated
PI3K	Buparlisib + Carboplatin / Lomustine	1	rGBM	NCT01934361 (Completed)	MTD: 100 mg/day with carboplatin, not determined with lomustine	-	-	-
PI3K	Paxalisib + Metformin + Ketogenic diet	2	rGBM and unmethylated ndGBM	NCT05183204 (Recruiting)	-	Yes	Ongoing	Ongoing
PI3K, PD-1	Pictilisib + MK-3475	1/2	GBM	NCT02430363 (Unknown status)	Unreported	No	Unreported	Unreported
AKT, mTORC1	Perifosine + Temsirolimus	1	rGBM	NCT02238496 (Completed)	Tolerable at 115 mg/week temsirolimus with 100 mg/day perifosine	-	-	-
AKT, PD-L1	Ipatasertib + Atezolizumab	1/2	GBM	NCT03673787 (Unknown status)	RP2D: 400 mg/day ipatasertib with 1200 mg atezolizumab every 3 weeks	No	Unreported	Unreported
mTORC1, CDK4/6	Everolimus + Ribociclib	Early 1	rGBM	NCT03834740 (Completed)	Unreported	Yes	Unreported	Unreported
mTORC1, CDK4/6	Everolimus + Ribociclib	1	Glioma	NCT03355794 (Completed)	Tolerable, RP2D: 120mg/m^2^ ribociclib with 1.2mg/m^2^ everolimus	-	-	-
mTORC1, VEGF	Everolimus + Bevacizumab + TMZ	2	GBM	NCT00805961 (Completed)	-	Yes	Median: 11.3 months	Median: 13.9 months
mTORC1, EGFR	Temsirolimus + Erlotinib	1/2	Glioma	NCT00112736 (Completed)	MTD: 15 mg temsirolimus with 150 mg erlotinib	Yes	PFS-6: 13.95%	Unreported
mTORC1, EGFR	Sirolimus + Erlotinib	2	rGBM	NCT00672243 (Completed)	-	Yes	Median: 6.9 weeks PFS-6: 3.1%	Median: 33.8 weeks
mTORC1, multi-kinases	Everolimus + Gefitinib	1/2	GBM	NCT00085566 (Completed)	Unreported	No	Unreported	Unreported
mTORC1, multi-kinases	Temsirolimus + Sorafenib	1/2	rGBM	NCT00329719 (Completed)	Considerable grade 3 + toxicity MTD: 200 mg sorafenib twice daily with 20 mg temsirolimus weekly	Yes	Median: 2.6 months for VEGFi-naïve group, 1.9 months for VEGFi-treated group Median: 17.1% for VEGFi-naïve group, 9.8% for VEGFi-treated group	Median: 6.3 months for VEGFi-naïve group, 3.9 months for VEGFi-treated group
mTORC1, multikinase	Sirolimus + Vandetanib	1	rGBM	NCT00821080 (Completed)	Tolerable, MTD: 200 mg vandetanib with 2 mg sirolimus	-	PFS-6: 15.8%	-
mTORC1, BCR-ABL tyrosine kinase, ribonucleotide reductase	Everolimus + Imatinib + Hydroxyurea	1	rGBM	NCT00613132 (Completed)	Unreported	-	-	-
mTORC1, AKT, 𝛾-secretase	Ridaforolimus + MK-2206 + MK-0752	1	rGBM	NCT01295632 (Completed)	Unreported	-	-	-
mTOR, proteasome	Nab-sirolimus + Marizomib	2	ndGBM	NCT03463265 (Completed)	-	Yes	Median: 1.7 months PFS-6: 10% PFS-12: 0%	Median: 6.7 months OS-12: 0%
Proteasome, VEGF	Bortezomib + Bevacizumab	2	rGBM	NCT00611325 (Completed)	-	Yes	Median: 2 months for EIAED arm, 2.5 months for non-EIAED arm PFS-6: 25% for EIAED arm, 28.6% for non-EIAED arm	Median: 8 months for EIAED arm, 6 months for non-EIAED arm
Proteasome, VEGF	Bortezomib + Bevacizumab + TMZ	1	rGBM	NCT01435395 (Completed)	Unreported	-	-	-
Proteasome, histone deacetylases	Bortezomib + Vorinostat	2	rGBM	NCT00641706 (Completed)	-	Yes	Median: 1.5 months for no surgery arm, 4.2 months for surgery arm PFS-6: 0% for no surgery arm, 29% for surgery arm	Median: 3.2 months for no surgery arm, 8.7 months for surgery arm
Proteasome	Disulfiram + Copper + TMZ + Lomustine + PCV	2/3	rGBM	NCT02678975 (Completed)	-	Yes	Median: 2.3 months	Median: 5.5 months OS-6: 44%
PARP	Talazoparib + Carboplatin	2	rGBM	NCT04740190 (Unknown status)	-	Yes	Unreported	Unreported
NSC	5-FU	1	rGBM	NCT01172964 (Completed)	Tolerable	-	-	-
NSC	5-FU, Leucovorin	1	rGBM	NCT02015819 (Completed)	Tolerable, RP2D: 150 × 10^6^ CD-NSCs	-	-	-
NSC	Irinotecan Hydrochloride	1	rGBM	NCT02192359 (Active, not recruiting)	Unreported	-	-	-
NSC	Adenovirus, TMZ	1	ndGBM	NCT03072134 (Completed)	Tolerable, RP2D: 1.50 × 10^8^ NSCs loading 1.875 × 10^11^ viral particles	Yes	Median: 9.1 months	Median: 18.4 months
MSC	5-FU	1/2	rGBM	NCT04657315 (Completed)	Tolerable	Yes	Median: >4 months	Median: not reached
MSC	Adenovirus	1	rGBM	NCT03896568 (Recruiting)	Ongoing	-	-	-

GBM, glioblastoma; rGBM, recurrent glioblastoma; ndGBM, newly diagnosed glioblastoma; RT, radiotherapy; TMZ, temozolomide; PCV, combination of procarbazine, lomustine, and vincristine; MTD, maximum tolerated dose; RP2D, recommended Phase 2 dose; RANO criteria, Response assessment in neuro-oncology criteria; PFS-3/6/12, 3/6/12 months progression free survival; OS-1/6/12, 1/3/6 year overall survival; EIAED, enzyme-inducing anti-epileptic drug

^1^Only in Phase 1 trials; ^2^Only in Phase 2 and Phase 3 trials

EGFR belongs to the ErbB receptor family of trans-membrane receptor tyrosine kinases. Aberrant EGFR activation has been observed in multiple cancers including gliomas, commonly driven by hyperactivating mutations and gene amplification. *EGFR* amplification leads to high protein expression of EGFR that activates a multitude of signalling cascades (including activation of PI3K/AKT, RAS/MAPK, and JAK/STAT pathways) contributing to tumorigenesis and progression [39, 40] (Fig. 1A). *EGFR* amplification is detected in more than 57% of primary GBM patients [41], with a predilection in the classical GBM subtype and only infrequently in other subtypes [11]. Besides amplification of *EGFR* and mutant ligands such as constitutively active EGFRvIII, *EGFR* can also be amplified in extra-chromosomal DNA (ecDNA) as amplicons that are capable of random integration into chromosomes and unequal segregation to daughter cells [42]. As such, ecDNA harbouring *EGFRvIII* would result in uncontrolled increase in oncogene copy number and intratumoral heterogeneity, preventing adequate drug targeting. There are several clinical trials exploring anti-EGFR strategy in GBM, such as dacomitinib, gefitinib, erlotinib, neratinib, and cetuximab (Table 1**and** Table 2). However, anti-EGFR targeted therapies have been less successful than expected due to acquired drug resistance and tumour heterogeneity, with only marginal increase in clinical benefits as monotherapy for GBM patients [43–48].

While activation of compensatory oncogenic signalling pathways such as the PI3K and MET pathways contributes to resistance against EGFR inhibitors in GBM, a significant limitation in current EGFR inhibitors is the specificity of its mechanism of action [49]. *EGFR* mutations commonly found in GBM are predominantly in the extracellular domain, limiting the efficacy of current first- and second-generation EGFR inhibitors such as dacomitinib, erlotinib and afatinib [40, 47, 50]. Though third-generation EGFR inhibitors, such as osimertinib, have been developed with improved binding to specific mutant EGFR, acquired mutations (C797S, G724S, L718Q) hinder the binding of inhibitors to EGFR and continue to limit the efficacy of third-generation EGFR inhibitors [51]. Unfortunately, even with the development of potent EGFR inhibitors, the oncogenic function of EGFR may still be retained in GBM cells through EGFR-PDGFRA receptor heterodimerization, suggesting the need for combinatorial treatment to effectively target EGFR activity [51].

To address the above-mentioned challenges of using EGFR inhibitors in GBM, novel therapeutics have been developed. Among which is ABT-414, an antibody-drug conjugate (ADC) that preferentially binds to overexpressed EGFR or EGFRvIII, thus conferring selective cytotoxic effect of monomethyl auristatin F to EGFR-amplified cells independent of EGFR signalling [52]. Despite exhibiting promising efficacy against both wildtype EGFR and EGFRvIII in cell lines and patient-derived xenografts [53], ABT-414 did not confer survival benefits in newly diagnosed or recurrent GBM patients in the INTELLANCE 2 trial [54, 55]. This is owing to the preferential loss of *EGFR*-amplification in resistant clones, which allows them to escape ABT-414 binding [56]. Another explanation is that ADCs are inefficient in penetrating the BBB into large tumours such as GBM [57, 58], which again highlights BBB penetrance as one of the biggest challenges in targeting GBM. In addition to harbouring EGFR mutations, cancer cells may also evade treatment by constitutively activating downstream effectors in an EGFR-independent manner. One such resistance mechanism in GBM is KRAS-driven hyperactivation of the MAPK signalling pathway, which can be facilitated by DDR1 overexpression [59]. To overcome KRAS-driven resistance to EGFR inhibitors, co-inhibition of EGFR and DDR1/BCR-ABL has previously demonstrated synergistic efficacy in retarding cell growth and inducing apoptosis in tumouroids of patient-derived recurrent GBM [60]. Overall, while targeting EGFR-driven tumorigenesis is a potentially effective therapeutic strategy, the unmet need for inhibitors with greater specificity towards GBM-associated EGFR mutations limits their current clinical actionability in GBM patients, and they should be administered in combination with other therapeutic agents to combat GBM resistance.

### Targeting signal transducer and activator of transcription 3 (STAT3) in GBM

Signal Transducer and Activator of Transcription 3 (STAT3) is another potential target of therapy for GBM. Acting downstream of multiple kinases and growth factor receptors, including but not limited to PDGFR, EGFR and IL-6, STAT3 is an important mediator of gliomagenesis through its role in modulating cancer cell survival, invasiveness, and immune evasion (Fig. 1B).

STAT3 is a transcription factor which modulates transcription of numerous genes involved in cell-cycle regulation and anti-cell death activity of the JAK/STAT3 pathway [61]. STAT3 is found to be constitutively active in 90% of human GBM tumours [62]. Persistent STAT3 activation occurs when there is hyperactivation in the upstream signalling cascade or defective downstream regulation, thus resulting in upregulation of several major oncogenic signalling pathways and contributing to tumorigenesis of multiple cancers, including GBM [63]. In GBM, STAT3 is reported to directly promote cell survival by enhancing expression of Bcl-2-like protein 1, driving the inhibition of cell death and promoting tumour cell proliferation [64]. Constitutively active STAT3 confers resistance to apoptosis by enhancing transcription of anti-apoptotic regulators including *Bcl-2*, *Bcl-XL* and *Mcl-1* (Fig. 1B) [62], whereas inhibition of STAT3 selectively induced apoptosis in WP1066-treated GBM cells by downregulating expression of anti-apoptotic genes and restoring BAX activity [65]. Furthermore, oncogenic STAT3 also confers resistance to autophagy by suppressing pro-autophagic pathways including Bcl-2/Beclin-1 and AMP-activated protein kinase ⍺ (AMPK⍺)/Unc-51-like kinase 1 (ULK1) signalling in GBM cells [66, 67]. Given the pivotal role of STAT3, targeting STAT3-dependent apoptosis and autophagy might be a promising strategy for sensitizing GBM cells to therapy-induced cell deaths.

STAT3 is also implicated in promoting cellular differentiation, namely in assisting the epithelial-mesenchymal transition (EMT) of radioresistant GBM by upregulating the expression of EMT markers such as MMPs, Rho and Rac [68, 69] (Fig. 1B). This promotes the migratory and invasive properties of glioma stem cells (GSCs), and are maintained via STAT3-mediated upregulation of the Notch pathway [70]. Importantly, activation of STAT3 signalling has been found to induce a switch from the less aggressive proneural to the more aggressive mesenchymal tumour subtype associated with chemoradiotherapy-resistance and recurrence in GBM [71]. Recently, it has been shown that depleting insulin-like growth factor binding protein 2 (IGFBP2) can lead to the sensitisation of STAT3-low expressing cells to STAT3 inhibitors, suggesting that targeting both the STAT3 and IGF-1R/IGFBP2 signalling axis is a promising therapeutic strategy for GBM [72]. To further contribute to the invasiveness of GSCs, constitutively active STAT3 can also enhance VEGF-mediated angiogenesis by promoting VEGF expression (Fig. 1B), facilitating neovascularisation and intracranial extension in GBM [73–75].

Therapeutics have thus been developed to target STAT3 as single agent or in combinations, and their potential use as anti-neoplastic drugs have been explored in preclinical settings. One class of inhibitors directly binds to and impedes STAT3 function. For instance, STA-21 targets the SH2 domain of STAT3, preventing STAT3 dimerization and suppressing stem cell properties in GSCs [76, 77]. Similar inhibitors that have been tested in preclinical studies include inhibitors of STAT3 phosphorylation, LLL12, an STA-21 analogue, LLL3, and an inhibitor of STAT3 dimerization, STX-0119 [78–80]. However, all these inhibitors have only demonstrated anti-cancer properties in preclinical models of GBM and have yet to advance into clinical studies.

Another therapeutic approach is to block STAT3 activation by attenuating the upstream signalling pathway in the form of JAK inhibitors. AG490 is a JAK2 inhibitor that has demonstrated efficacy in mitigating STAT3 activity through downregulation of STAT-regulated genes *MMP2* and *MMP9* in GBM cell lines, albeit exhibiting limited anticancer effect in vivo [62, 65, 81, 82]. WP1066, a more potent second-generation analogue of AG490, demonstrated compelling in vivo anti-tumour effect against GBM as well as in patient-derived GSCs [82, 83]. Following promising preclinical results, a Phase 1 trial was conducted to determine the safety of WP1066 in patients with recurrent GBM [84] (Table 1). However, given that subjects in this Phase 1 trial were heavily pre-treated recurrent GBM patients, WP1066 monotherapy did not significantly improve patients’ PFS as patients may have developed several mechanisms of general drug resistance [84]. Moving forward, the potential therapeutic effect of WP1066 and radiotherapy in treating newly diagnosed GBM patients will be evaluated in a planned Phase 2 trial, at the maximum tolerated dose/maximum feasible dose of 8 mg/kg identified in the Phase 1 trial [84].

A more recent approach to target STAT3 is through the use of oligonucleotide therapeutics. This approach includes antisense oligonucleotides (ASO) such as AZD9150, GQ-ODN and decoy oligonucleotide [85–91]. Though oligonucleotide therapeutics have achieved success in other cancer types and proceeded on to clinical trials (NCT01839604) [85, 86, 89], BBB penetration and drug delivery still remain a challenge for brain tumours such as GBM. Thus, while nucleic acid therapeutics show promise in targeting STAT3 in cancer, clinical inhibition of STAT3 in GBM is still dependent on the development of effective pharmacological agents which can cross the BBB.

### Targeting the PI3K/AKT/mTOR signalling pathway in GBM

Given its involvement in tumour development and progression, the phosphoinositide 3-kinase (PI3K)/AKT/mammalian target of rapamycin (mTOR) pathway has emerged as a promising therapeutic target in GBM. The PI3K/AKT/mTOR signalling network is known to be activated in nearly 90% of GBM patients [11], making it a potentially beneficial therapeutic target. This pathway is crucial for cell survival, proliferation, and angiogenesis, all of which contribute to the aggressive nature of GBM. By targeting this pathway, it is possible to disrupt the aberrant signalling cascades that drive tumour growth and improve treatment outcomes.

Several mechanisms contribute to the hyperactivation of the PI3K/AKT/mTOR pathway in GBM. As discussed in the previous section, upstream receptor amplification, such as EGFR and PDGFR, is a typical mechanism leading to PI3K/AKT/mTOR pathway hyperactivation in GBM. These amplifications enhance ligand binding and, as a result, activate the PI3K/Akt/mTOR signalling cascade.

Phosphatase and Tensin Homolog *(PTEN*), which is located on the q arm of chromosome 10 (10q), functions as a negative regulator of the PI3K/Akt/mTOR signalling pathway (Fig. 1C), and is deleted in 90% of primary GBM due to a loss of heterozygosity of chromosome 10 [11, 92] [11, 92]. PTEN function can also be lost due to homozygous and hemizygous deletion of the gene [11], as well as poor stability of the mutant protein [93]. PTEN deficiency or dysfunction results in sustained stimulation of PI3K/AKT/mTOR signalling in either instances, resulting in poorer prognosis in PTEN-deficient GBM patients [94, 95].

In addition to PTEN loss, mutations in the PI3K complex can contribute to PI3K/AKT/mTOR pathway hyperactivation in GBM. Activating somatic mutations in phosphatidylinositol-4,5-bisphosphate 3-kinase catalytic subunit alpha (*PIK3CA*) or phosphoinositide-3-kinase regulatory subunit 1 (*PIK3R1*) genes are frequent alterations that disrupt the conformation of the p110⍺ catalytic subunit, resulting in constitutive PI3K activation [96, 97]. To target hyperactivation of PI3K, pan-PI3K inhibitors, isoform-selective PI3K inhibitors and dual PI3K/mTOR inhibitors have been developed. Alpelisib, a p110⍺-selective inhibitor licensed by the FDA for the treatment of *PIK3CA*-mutated breast cancer, has demonstrated preferential inhibition of proneural GSCs [98]. Thus far, only a few pan-PI3K inhibitors (buparlisib, pilaralisib and sonolisib) and dual PI3K/mTOR inhibitors (paxalisib, dactolisib, voxtalisib, PQR309) have been evaluated in clinical trials for the treatment of GBM (Table 1**and** Table 2), among which, buparlisib is the most commonly studied. Despite demonstrating strong efficacy in vitro and greater BBB permeability, such positive results did not translate to clinical efficacy when buparlisib was investigated as a single agent and in combination with the standard radio-chemotherapy [99–101]. Paxalisib, a dual PI3K/mTOR inhibitor specifically developed for the treatment of GBM, has demonstrated favourable safety profile and promising efficacy as a first-line treatment in a Phase 2 trial, and is being further investigated in patients with newly diagnosed or recurrent GBM as a part of the AGILE GBM trial [102, 103]. However, recent preclinical studies have revealed that tumour cells in GBM and other cancers may circumvent PI3K inhibition by inducing insulin feedback as a resistance mechanism to reactivate PI3K-mTOR signalling, suggesting that PI3K inhibitors may need to be coupled with anti-hyperglycemic therapies such as metformin to increase treatment efficacy [104, 105]. Correspondingly, a clinical trial is therefore underway to assess the clinical value of this strategy by combining paxalisib with metformin and a ketogenic diet (NCT05183204) (Table 2).

While less common and less characterized, hyperactivation of AKT and mTOR can occur downstream of the pathway in GBM without significant mutations [106]. Upstream signalling components, such as RTKs and PI3K can be dysregulated, resulting in increased activation of AKT or mTOR without necessity for direct mutations in these genes. As a critical central regulator of multiple oncogenic signals, aberrant activity of mTOR and downstream effectors including S6K and 4EBP1 is significantly higher in GBM in comparison to low grade glioma (Fig. 1C), implying that AKT and mTOR effector activity may be emerging as novel prognostic markers of glioma malignancy [107, 108].

The only AKT-specific inhibitor that has been tested in GBM patients is the allosteric inhibitor, perifosine. However, like most other investigational drugs, efficacy of perifosine was not observed in GBM patients [109]. In recent years, more AKT inhibitors are developed and tested pre-clinically as candidate drugs for GBM therapy. MK2206, a new allosteric inhibitor of AKT, was found to potentially sensitize GBM spheroids to TMZ treatment and radiotherapy, warranting further investigations into its clinical prospects for GBM patients [110].

On the other hand, as downstream effectors of AKT, mTOR complexes are more attractive as therapeutic targets for clinical investigation. Among mTOR inhibitors, the most commonly investigated drugs in GBM are sirolimus and its analogues, including everolimus and temsirolimus, which are collectively known as rapalogues (Fig. 1C). However, they have shown limited efficacy in clinical trials as single agents or in combination with the current standard of care (Table 1). Sirolimus only exhibited anti-GBM effects in PTEN-deficient GBM patients as a single therapeutic agent, and has demonstrated limited additional benefit when combined with EGFR inhibitor, erlotinib [111, 112]. Notably, rapalogues preferentially inhibit mTORC1 as opposed to mTORC2 [113]. On the contrary, vistusertib, an inhibitor of both mTORC complexes, demonstrated therapeutic effects in sensitizing stem-like GBM cells to radiation both in vitro and in vivo [114]. Such promising preclinical results have encouraged the conduct of an ongoing Phase 1 trial in recurrent GBM (NCT02619864). Another dual mTORC1/2 inhibitor, onatasertib has demonstrated potential antitumour activity in patients with advanced solid tumours including GBM [115]. AZD8055, dual mTORC1/2 inhibitor, additionally warranted further clinical investigation (NCT01316809) after demonstrating the induction of autophagy and autophagy-regulated Notch1 degradation in GBM cell lines [116]. Importantly, AZD8055 also demonstrated synergistic inhibitory effect and improved survival with TMZ in orthotropic xenografts [117]. These collectively suggest that dual inhibition of both mTOR complexes is essential in targeting the PI3K/AKT/mTOR axis in GBM as opposed to only targeting mTORC1 which could result in compensatory activation of mTORC2 and hence limited clinical efficacy.

Studies have additionally investigated how GBM cells can circumvent therapy resistance induced by the PI3K/AKT/mTOR pathway. Specifically, Vehlow and team found that AKT increased pro-survival signalling in GBM by associating with DDR1, 14-3-3 and Beclin-1 in a complex [118]. Inhibition of DDR1 in the complex suppressed the pro-survival AKT and mTOR signalling pathway and resensitized GBM cells to radio- and chemotherapy by activating autophagy [118]. Concurrent attenuation of the PI3K and EGFR signalling axis through DDR1 and EGFR inhibitors respectively may also revert KRAS-induced hyperactivation in recurrent GBM [60]. These studies highlight DDR1 as a promising and upcoming target in inhibiting the PI3K axis in GBM. While DDR1 inhibition is a novel therapeutic strategy against recurrent GBM in vitro, further validation of its in vivo and clinical efficacy is warranted, and the development of more DDR1-specific inhibitors holds promise as targeted therapies against GBM.

### Targeting IDH1/2-associated vulnerabilities in GBM

*IDH1/2* mutation status is a prognostic marker used to differentiate between astrocytoma and GBM as *IDH* mutations are associated with more optimistic prognosis. Patients harbouring *IDH*-mutant gliomas exhibit better survival than *IDH* wildtype gliomas in GBM (31 months vs. 15 months) and anaplastic astrocytoma (65 months vs. 20 months) [119]. Despite its association with lower-grade gliomas, *IDH* mutations are often observed in secondary GBM (73%) as well [120], suggesting that lower-grade *IDH*-mutant gliomas are prone to malignant progression and recurrence as higher-grade gliomas [121]. Targeting vulnerabilities in *IDH*-mutant gliomas is thus a viable strategy to mitigate the risks of recurrence in patients.

*IDH1/2* mutation is associated with the induction of multiple mechanisms that drive gliomagenesis. IDH1 plays a major role in metabolic pathways by metabolizing isocitrate to produce alpha-ketoglutarate (⍺-KG). *IDH* mutation in glioma often occurs at arginine residues that are responsible for isocitrate binding (R132 for *IDH1*, R140 or R172 for *IDH2*) [122]. Heterozygous missense mutations, which are the major forms of *IDH1* mutations observed in *IDH*-mutant gliomas, generate mutant IDH1 that converts isocitrate into the oncometabolite D-2-hydroxyglutarate (D-2-HG) in place of ⍺-KG [123]. Accumulation of D-2-HG in glioma cells triggers multiple aberrant cellular processes, including epigenetic modifications which contribute to metastasis progression through induction of oncogenes activation and silencing of tumour suppressor genes [124, 125].

A key epigenetic characteristic of *IDH*-mutant glioma is the presence of hypermethylation. Hypermethylation in *IDH*-mutant GBM cells is mostly mediated by DNA methylation and histone methylation. Due to structural similarity, oncometabolite D-2-HG competitively inhibits ⍺-KG-dependent ten-eleven translocation (TET) demethylase, decreasing the functional activity of TET demethylase which is responsible for reversing DNA methylation [126–128] (Fig. 1D). Consequently, DNA methylation may be extended in glioma cells to establish glioma CpG island methylator phenotype (G-CIMP) [129], and potentially leads to irreversible epigenetic alterations which commit glioma cells to oncogenesis, such as the silencing of tumour suppressive microRNA, miR-148a [130], as well as activation of oncogene, *PDGFRA* [131]. Furthermore, D-2-HG inhibition of TET demethylase in IDH-mutant glioma cells has also been shown to maintain stemness. Inhibition of TET demethylase in *IDH1*-mutant astrocytes resulted in upregulation of stem cell marker, Nestin [129], whereas restoration of TET2 expression in GBM cells upregulated genes crucial for neural differentiation, such as brain fatty acid-binding protein (BFABP) and Mash1 [132]. Taken together, D-2-HG-mediated hypermethylation in *IDH*-mutants contributes to gliomagenesis by triggering downstream epigenetic alterations of oncogenes and impairments in cellular differentiation.

One clinical significance of *IDH*-mutant-induced histone methylation is the silencing of *MGMT*, a gene involved in DNA damage repair. Methylation at two key differentially methylated regions (DMRs) of the *MGMT* promoter, DMR1 and DMR2, has been identified as contributing factors of *MGMT* silencing in GBM patients [133, 134], which accounts for approximately 45% of patients [135]. While the mechanism of methylation at DMR2 has yet to be elucidated, the positive association between *IDH1* mutation and high *MGMT* promoter methylation has been shown in xenografts and GBM patients [136, 137]. This is further supported by the better therapeutic effects of TMZ for *IDH1*-WT glioma patients with *MGMT* promoter methylation over *IDH1*-mutants [138]. Given that the G-CIMP is highly enriched in *IDH1*-mutant proneural GBM [139], as well as the positive association between *MGMT* methylation and G-CIMP in GBM tumours [41], the role of *IDH1* mutations in *MGMT* silencing may be related to *IDH*-mutant-induced histone methylation. These findings show that *IDH1* mutation and high *MGMT* methylation may offer prognostic value as paired predictive biomarkers.

In *IDH*-mutant GBM cells, the oncometabolite D-2-HG interferes with the homologous recombination (HR) pathway by masking H3K9 trimethylation signal through hypermethylation, preventing the recruitment of homology-dependent repair factors to the site of double strand break (DSB), **i**ncluding ATM and histone demethylase KDM4B [140, 141]. This results in a state of ‘BRCAness’, where the *IDH*-mutant GBM cells exhibit a phenotype similar to *BRCA*-mutant cancer cells with impaired HR pathway. Similarly, IDH-mutant glioma cells demonstrate synthetic lethality with PARP inhibition, and are hence vulnerable to PARP inhibitors [140, 141]. (Fig. 1D). By inhibiting the DNA repair pathway, PARP-inhibitors enhance cytotoxicity of chemotherapy by inducing excessive accumulation of DNA damage which results in elevated apoptosis observed in PARP-inhibitor treated *IDH1* mutant GBM cells [142].

Currently, clinically investigated PARP inhibitors in GBM include olaparib, niraparib, BSI-201, BGB-290, veliparib, fluozoparil, and NMS-03305293 (Table 1**and** Table 2). Olaparib was granted FDA approval for the treatment of *BRCA*-mutated advanced breast, ovarian and pancreatic cancer, and was recently investigated in the application for GBM. In the Phase 1 OPARATIC trial, olaparib was detected at radiosensitizing concentrations in all recurrent GBM tumour specimens, demonstrating its ability to cross the BBB [143]. Promising therapeutic profile observed encouraged ongoing trials to further investigate the clinical potential of olaparib in *IDH1/2*-mutant GBM [144] as well as *tumour protein P53* (*TP53*) mutant GBM (NCT05432518). While olaparib did not meet the pre-specified response-based threshold to proceed with a Phase 3 trial in *IDH*-mutant glioma patients, a subset of patients demonstrated prolonged stable disease, suggesting that olaparib may still be clinically useful as a form of maintenance therapy for a subset of GBM patients [144]. An alternative PARP inhibitor, niraparib was found to exhibit better tumour exposure and sustainability than olaparib, and was tolerable when administered in combination with TMZ, though the two drugs demonstrated no synergy [145, 146]. Previous studies have also shown that PARP inhibitors exhibit radiosensitizing effects by inhibiting the base excision repair pathway [147–150]. The combination of niraparib and radiotherapy is thus being evaluated in three ongoing studies (NCT04221503, NCT04715620, NCT05076513) (Table 1). In the Phase 1 trial for GBM patients, the combination demonstrated promising efficacy and favourable safety characteristics [151].

However, tumour-suppressing effect of PARP inhibitor is low in *IDH1/2-*wildtype gliomas. In *IDH1/2*-wildtype cells, functional BRCA1/2 can be recruited to the site of DNA damage to carry out HR and allow cells to bypass the damaged site [152]. To confirm this finding, an ongoing clinal study is investigating the efficacy of the combination of PARP inhibitor NMS-03305293 and TMZ in *IDH*-wildtype GBM (NCT04910022). While the trial is currently ongoing, the results would provide justification for stratifying patients according to the mutation status of *IDH* when utilizing PARP inhibition as a therapeutic strategy for GBM.

Given that the neomorphic activity of IDH promotes oncogenesis in *IDH*-mutant GBM cells through the production of oncometabolite D-2-HG, IDH inhibitors such as ivosidenib and vorasidenib have also been developed to block the downstream production of D-2-HG. Notably, IDH inhibitors have shown promising clinical efficacy in only *IDH*-mutant low-grade glioma (LGG) patients. Therapeutic benefits of vorasidenib and ivosidenib in treatment IDH-mutant LGGs have been demonstrated in multiple trials, especially in the INDIGO trial, in which vorasidenib significantly improved PFS in LGG patients [153–156]. Importantly, promising results from the INDIGO trial has granted vorasidenib priority review by the FDA. On the other hand, the only reported clinical case of an IDH-mutant GBM patient treated with an IDH inhibitor is a recurrent GBM patient treated with ivosidenib in a Phase 1 clinical trial [153, 157]. Given the high proportion of *IDH*-mutated diffuse astrocytoma, CNS WHO grade 4, in secondary GBM, it would be advantageous to potentially extend the IDH inhibitor trial cohort to GBM patients so as to evaluate the therapeutic value of IDH inhibitors in secondary GBM.

### Targeting protein clearance in GBM: proteasomes

**Fig. 2:**
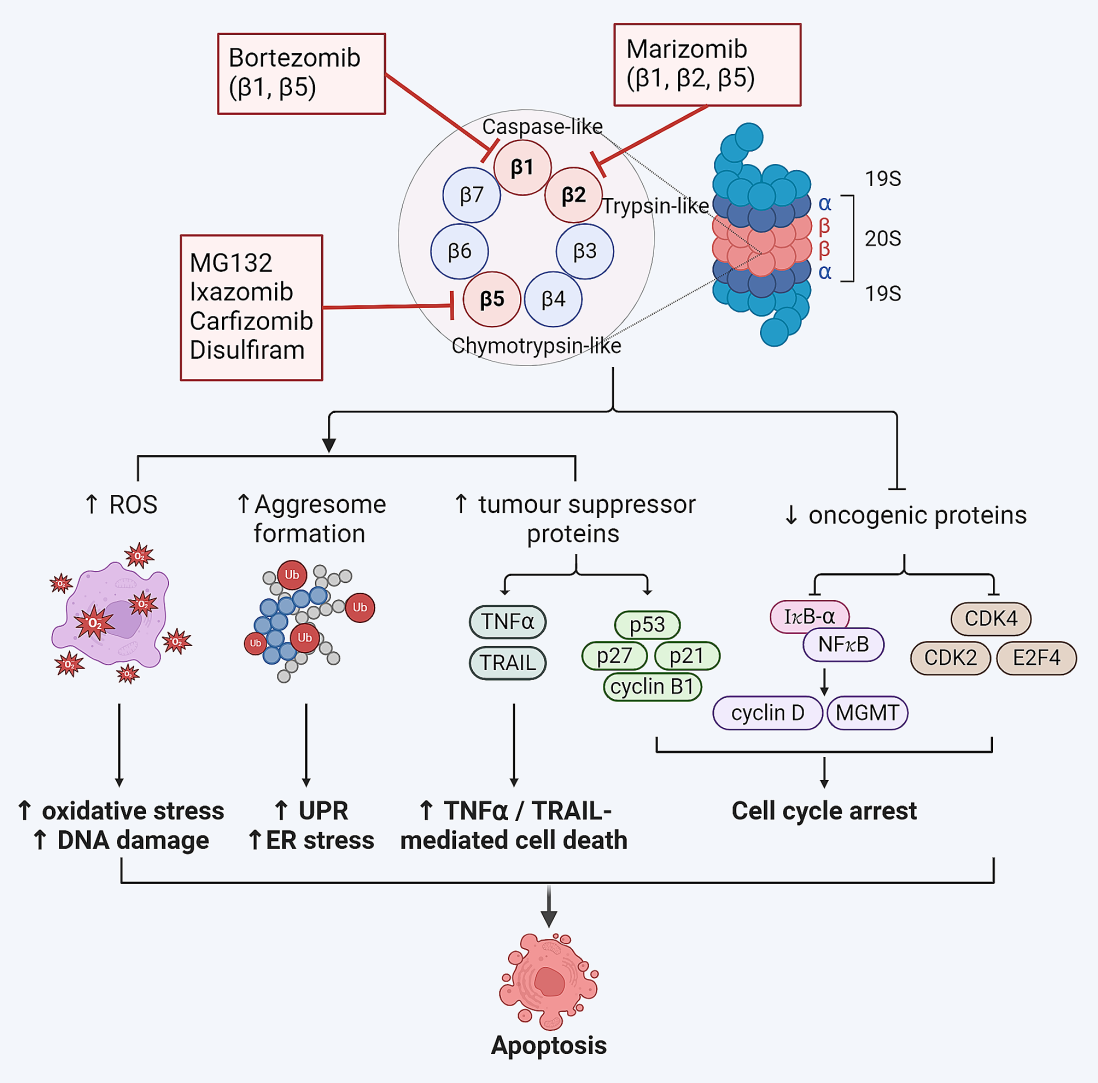
Schematic of 20S proteasome inhibitors and their targets. Most proteasome inhibitors (in red boxes) target the common β5 chymotrypsin-like site of the 20S CP as it is the most important active site for protein breakdown. MG132 is the first proteasome inhibitor developed. The first-in-class proteasome inhibitor bortezomib and other second-generation proteasome inhibitors were later developed with enhanced potency and specificity for more active sites. Functional inhibition of the proteasome results in activation of pro-apoptotic and suppression of oncogenic pathways

Proteasomes are responsible for the degradation of unwanted or damaged intracellular proteins, as well as the regulation of proteins that are involved in cell cycle and apoptosis. Proteasomes carry out the degradation of proteins through protein ubiquitination and proteolysis, together with other components in the ubiquitination-proteasome pathway (UPP) [158]. Oncogenic addiction to high proteasome levels has been observed in a multiple malignancies as an adaptation to high protein homeostasis in rapidly proliferating cancer cells [159]. UPP is also exploited in cancer cells to downregulate tumour-suppressor proteins such as p21, p27 and p53, as well as to activate oncogenic targets including NF-𝝹B, essentially contributing to carcinogenesis [160, 161]. The selective potency of proteasome inhibition in cancer cells supports the dependency on proteasome functions in cancer cells, implying that proteasome inhibitors may be used as a targeted therapeutic agent [162]. Inhibition of the UPP in GBM cells results in a plethora of biological effects, including the stimulation of oxidative stress, ER stress, receptor-mediated cell death and cell cycle arrest [163–168] (Fig. 2). This knowledge has led to bortezomib being the first proteasome inhibitor to be approved for the treatment of multiple myeloma [169]. The success of bortezomib has spurred many studies to investigate the potential application of proteasome inhibition in other malignancies, including GBM.

Most proteasome inhibitors target the β5 catalytic subunit of the 20S core particle (CP) of the 26S proteasome, which is responsible for chymotrypsin-like proteolytic activities (Fig. 2). Among these proteasome inhibitors, bortezomib is the most investigated drug in clinical studies. In one study, bortezomib was found to be more beneficial for *MGMT*-methylated patients compared to unmethylated patients in terms of PFS (24.7 months vs. 5.1 months) and overall survival (OS) (61 months vs. 16.4 months) [170]. However, promising results from this study were contradicted by other in vivo studies, in which bortezomib exhibited limited BBB penetration, and thus had limited efficacy in animal models [171]. Consequently, an alternative 20S proteasome inhibitor with greater BBB penetrance, marizomib, was developed [171, 172]. Marizomib exerts antitumour effects in GBM by inducing caspase 9-dependent apoptosis [173], and it could potentially achieve synergistic effect with TNF-related apoptosis-inducing ligand (TRAIL) receptor agonist [174]. Despite having promising pre-clinical results and passing two clinical trials, the efficacy of marizomib in the Phase 3 trial was disappointing, though it could be attributed to the lack of patient stratification for *MGMT* promoter methylation status in the trial [175–177] (Table 1**and** Table 2).

Disulfiram is another proteasome inhibitor that has potential therapeutic effect in treating GBM. Disulfiram is an FDA-approved acetaldehyde dehydrogenase inhibitor originally approved for the treatment of alcoholism. It was later discovered that disulfiram can additionally inhibit chymotrypsin-like activity by forming a proteasomal-inhibitory complex with tumour cellular copper, initiating apoptosis in copper-rich cancer cells [178, 179] (Fig. 2). Further functional analysis indicated that disulfiram inhibits protein turnover in GBM cells by targeting the p97/NPL4 pathway, which is essential for the processing of ubiquitylated proteins [180–182]. Clinical trials are therefore underway to investigate the efficacy of disulfiram together with copper gluconate to recreate the copper-rich environment necessary for the inhibition of the chymotrypsin-like proteases in GBM patients (Table 1**and** Table 2). While treatment with disulfiram, copper gluconate and TMZ exhibited manageable safety profiles and preliminary clinical benefits, combination therapy including other alkylating agents such as lomustine induced severe adverse events in patients, limiting the use of disulfiram in combination with alternative standard of care for GBM patients apart from TMZ [183–186].

### Targeting fusion genes in GBM

Recent advances in sequencing technologies, such as fluorescence in situ hybridization and Next-Generation Sequencing, have paved the way for robust characterization of the genomic landscape in GBM, resulting in the discovery of novel oncogenic fusion genes. Genomic and molecular studies have discovered many fusion genes in GBM, including fibroblast growth factor receptor (*FGFR*) fusions, anaplastic lymphoma kinase (*ALK*) fusions, *EGFR* fusions, and neurotrophic tyrosine receptor kinase (*NTRK*) fusions [187–191]. *FGFR* fusions are the most common and well-studied fusion gene in GBM, accounting for up to 8.33% of GBM patients [190–192]. *EGFR* fusions are the second most common fusion in GBM (4%) [188]. *ALK* fusions are more prevalent in paediatric GBMs, with only 1.9% found in adult GBMs [193]. *NTRK* fusion on the other hand are relatively more uncommon in GBM (1.2%) [193]. To date, several clinical cases have reported effective application of inhibitors for treatment of gliomas harboring fusion genes, including lorlatinib for the treatment of *SPECC1L-ALK* fusion harbouring paediatric high-grade glioma and larotrectinib against *EML4-NTRK3* positive recurrent GBM [194, 195]. As *FGFR-TACC* fusions, specifically *FGFR3-TACC3*, are the most common gene fusions reported in GBM, the discussions in this section will be focused on the therapeutic value of targeting FGFR3-TACC3 in GBM.

FGFR3-TACC3 originates from tandem duplication of the *FGFR3* and *TACC3* genes on 4p16.3 [191]. The coiled-coil domain at the C-terminus of TACC3 in the oncogenic chimeric protein facilitates kinase transphosphorylation and localization of FGFR3-TACC3 to the mitotic spindle, where it disrupts chromosomal segregation, resulting in chromosome instability (CIN) and aneuploidy [189]. Physiologically, expression of *FGFR3* is negatively regulated by the binding of miR-99a to the 3’-UTR of *FGFR3* transcripts [191]. This regulation is absent in *FGFR3-TACC3* positive GBM cells due to truncation of the 3’-UTR-containing C-terminal in the fusion transcript [191]. Without posttranscriptional regulation by miR-99a, *FGFR3-TACC3* fusion is overexpressed in GBM cells, resulting in production of the hyperactive chimeric oncoprotein. Interestingly, the fusion genes and either *IDH1/2* mutations or *EGFR* amplification were found to be mutually exclusive [190, 191]. This finding has refined the selection criteria for multiple clinical trials investigating therapeutic effects of FGFR inhibitors in GBM patients.

To date, a handful of FGFR inhibitors are being investigated in clinical studies to target *FGFR-TACC*-positive GBM. Erdafitinib (JNJ-42,756,493), a pan-FGFR selective inhibitor, has demonstrated survival benefits in mice bearing FGFR-TACC gliomas [189]. Clinically, erdafitinib showed antitumour activity in recurrent GBM in two Phase 1 studies [189, 190, 196]. Hence, a Phase 2 trial studying erdafitinib on *IDH*-wild type gliomas with *FGFR-TACC* gene fusion is now ongoing (NCT05859334). Another FGFR inhibitors, pemigatinib, has demonstrated promising results in the FIGHT-207 trial in solid tumours including GBM, and is now being applied in the GBM-focused FIGHT-209 trial [197, 198]. Infigratinib is also a promising FGFR inhibitor that achieved partial response or stable disease in 34.6% of recurrent GBM patients [199]. Numerous other FGFR inhibitors that have been investigated in GBM are fexagratinib, ponatinib, anlotinib and futibatinib, reflecting the clinical potential of FGFR inhibitors in GBM [200–203]. Taken together, targeting oncogenic fusion genes in GBM provides a personalized treatment avenue that holds great promise, especially for the subset of gliomas with druggable kinase fusion, despite their low occurrence rate.

## Emerging strategies to target cancer cells in GBM

**Fig. 3:**
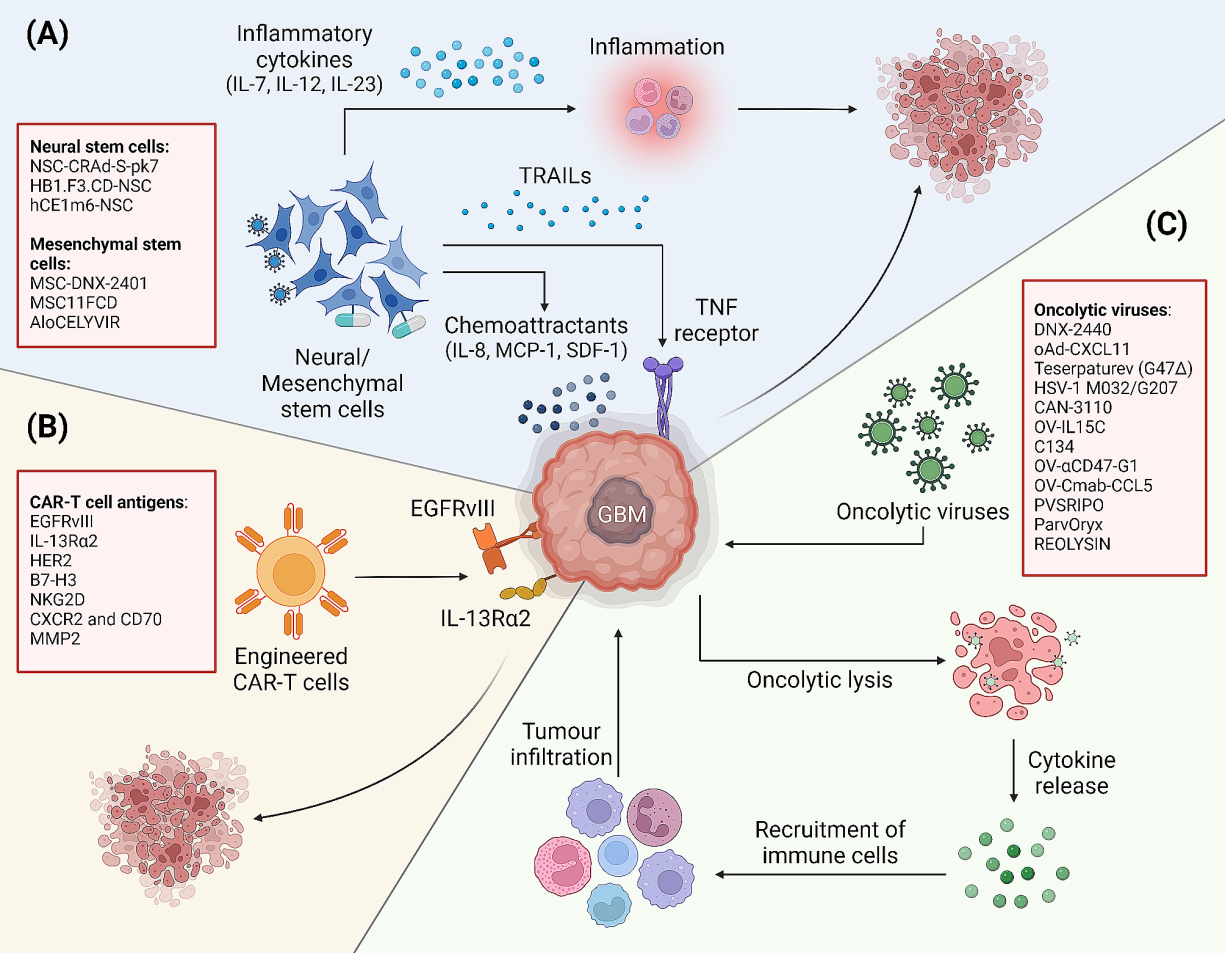
Schematic representation of alternate treatment modalities investigated in GBM. **(A)** Neural and mesenchymal stem cells (NSCs and MSCs) exhibit chemotactic migratory capabilities and will migrate to GBM tumours where elevated levels of chemoattractants are present. NSCs and MSCs elicit cell death in GBM tumours by secreting proinflammatory chemokines and TRAILs into the tumour microenvironment. Additionally, NSCs and MSCs can be loaded with oncolytic viruses, or co-administered with drugs to facilitate specific delivery of these anti-cancer agents to tumours. NSCs and MSCs developed against GBM tumours are listed in the red box. **(B)** Chimeric antigen receptor (CAR) T cells (in red boxes) have been engineered to bind to tumour-associated receptors commonly expressed in GBM tumours, such as EGFRvIII and IL-13Rα2, allowing for the induction of tumour-specific T cell cytotoxicity. **(C)** Oncolytic virotherapy has been developed for the specific targeting of GBM tumours by inducing oncolytic lysis of the tumour cells. During which, immunostimulatory cytokines are released into the tumour microenvironment. Consequently, various immune cells are trafficked to the tumour to mount an anti-tumour immune response. Various oncolytic viruses have been engineered and are investigated for the treatment of GBM (in the red box)

Apart from targeted pharmacological agents, alternate modalities for targeting GBM tumours have been investigated. This includes the use of oncolytic viruses which have been shown to exhibit antineoplastic properties. Recently, emerging technologies have been developed to treat the malignancy, such as leveraging on cell-based therapies, trafficking of immune cells to the tumour, and using pulsed ultrasound to transiently disrupt the BBB, thereby enhancing the delivery of targeted therapies which would otherwise be unable to penetrate the BBB [204, 205]. This section focuses on the developments and emergence of alternative treatment modalities to specifically target the receptors presented on cancer cells in GBM tumours. Specifically, progress in neural and mesenchymal stem cell therapy, chimeric antigen receptor (CAR) T-cell therapy, and oncolytic virotherapy will be discussed **(**Fig. 3**)**.

### Stem cell therapy in GBM: neural stem cells (NSC) / mesenchymal stem cells (MSC)

The discovery of neural stem cells (NSCs) and mesenchymal stem cells (MSCs) has led to the development of novel cell-based therapies to overcome the challenge of BBB penetration and achieve better management of GBM. NSCs are specific groups of multipotent stem cells found in the brain, with the potential to self-renew and differentiate into astrocytes, neurons, and oligodendrocytes [206]. Advancement in somatic cell reprogramming has further allowed NSCs to be generated through transdifferentiation, allowing the conversion of human skin fibroblast into “human-induced Neural Stem Cells (hi-NSCs)” [207, 208] This technique has further been applied in fibroblasts derived from GBM patients, permitting the establishment of patient-specific hi-NSCs [209]. On the other hand, MSCs are multipotent stem cells responsible for the generation of differentiated cells of the mesenchymal lineage and can be found in multiple tissue types including the bone marrow, adipose, muscle and umbilical cord [210].

Apart from their ability to cross the BBB, NSCs and MSCs are attractive treatment modalities due to their unique glioma-tropic migration capabilities [211–213]. Both NSCs and MSCs express cell surface markers and secrete cytokines that facilitate the chemotactic migration through normal tissues and preferentially home to the tumour **(**Fig. 3A**)**. Specifically, NSCs and MSCs express higher levels of chemokine receptors CXCR1, CXCR2, CXCR4 and CCR2 to facilitate the chemotactic migration of the cells to GBM tumours exhibiting elevated levels of IL-8, SDF-1 and MCP-1 [214–216].

hi-NSCs and MSCs can be manipulated to achieve specific aims, including but not limited to immunomodulation and drug delivery. For instance, hi-NSCs have been engineered to maintain an anti-tumour microenvironment by secreting inflammatory mediators including IL-7, IL-12 and IL-23, thereby recruiting immune cells to inhibit the growth of gliomas **(**Fig. 3A**)** [211, 217, 218]. Several studies have also evaluated the therapeutic effect of TRAIL-producing NSCs in targeting GBM tumours. TRAIL-producing hi-NSCs induced cell death selectively in GBM cells via caspase-mediated apoptosis, successfully mitigating GBM development and extended median survival in human GBM xenografts **(**Fig. 3A**)** [212, 219–221].

Given their ability to penetrate the BBB, NSCs and MSCs can also serve as vectors in enzyme/prodrug-based and oncolytic virus-based drug delivery systems **(**Fig. 3A**)**. The enzyme/prodrug-based system can be achieved by modifying the NSCs/MSCs to produce enzymes that convert prodrugs into their active therapeutic forms. An example of such a system is the FDA-approved cytosine deaminase (CD)-expressing clonal human NSC line, HB1.F3.CD, engineered to home to gliomas and convert prodrug 5-fluorocytosine (5-FC) to the active chemotherapeutic 5-fluorouracil (5-FU) [222]. Clinical assessment of this system demonstrated specific NSC and 5-FU localization in the brain tumour and was well tolerated by patients [223]. The safety profile of 5-FU-releasing NSCs in combination with leucovorin in high-grade gliomas was further established, and its efficacy is currently under investigation [224]. Carboxylesterase-releasing NSCs are also currently in clinical trial to investigate their ability to sensitize recurrent high-grade gliomas to irinotecan hydrochloride by converting the prodrug irinotecan into its active metabolite, SN-38 (NCT02192359) (Table 2). Development and clinical assessments of similar enzyme/prodrug-based systems in MSCs are also currently underway (NCT04657315) [225].

The application of NSCs and MSCs as carriers has also recently been extended into the delivering of adenovirus to overcome shortcomings in virotherapy such as the activation of host immunological response and poor biodistribution [226]. The most notable applications are NSCs loaded with CRAd-S-pk7 (NSC-CRAd-S-pk7), a glioma-restricted oncolytic adenovirus, which could improve viral biodistribution in mice brains, enhance inhibition of GBM tumour growth and improve median survival by 50% compared to viral treatment alone [227–229]. The NSC-CRAd-S-pk7 system exhibited favourable safety profiles in 12 newly diagnosed malignant glioma patients, with a median progression-free survival of 9.1 months and OS of 18.4 months [230]. Synergy between NSC-CRAd-S-pk7 with standard of care treatments was also observed in in vivo models of GBM, increasing survival by approximately 46% [228]. The safety profile of this approach was also determined, with a reported clearance of NSC and viral components from various regions of patients’ brains 4–24 months after treatment, suggesting that multiple administration of the NSCs is tolerable in patients whilst simultaneously achieving greater therapeutic benefit [230]. Following the promising development of NSC-CRAd-S-pk7, MSCs loaded with oncolytic adenoviruses have since been engineered and clinical trials are currently undergoing to evaluate their value as prospective treatment modalities against GBM (NCT03896568, NCT04758533). Taken together, the evidence has suggested that hi-NSCs and MSCs are promising and novel therapeutic delivery vectors that provide specific drug localization, improving clinical response in GBM patients.

### Chimeric antigen receptor (CAR) T-cell therapy in GBM

Recently, the use of chimeric antigen receptor (CAR) T-cells as a form of cell-based immunotherapy and treatment strategy for GBM patients has been gaining interest. To develop CAR T-cells, chimeric antigen receptors, comprising of extracellular domains which recognise specific epitopes frequently presented on the tumours and intracellular domains necessary for T-cell activation, are engineered into isolated T-cells. This allows for the CAR T-cells to home specifically to the tumour sites to exert their cytotoxic effects, bypassing the need for MHC antigen presentation.

To date, several CAR T-cells targeting various antigens frequently reported in GBM tumours have been generated and are being evaluated as viable treatment modalities in the clinics. Notably, the most common tumour-associated antigens are EGFRvIII and interleukin 13-receptor α 2 (IL-13Rα2) **(**Fig. 3B**)**. However, clinical trials of CAR T-cells targeting either EGFRvIII or IL-13Rα2 while tolerated in patients, did not present durable response in patients [231–233]. In particular, only one patient demonstrated a complete response to IL-13Rα2-targeting CAR T-cell therapy before tumour recurrence [234]. Hence, there have been recent identification of alternative target antigens for which CAR T-cells have been developed against, including HER2 and B7-H3 **(**Fig. 3B**)** [235–238]. Interestingly, recent preclinical investigations have identified the natural compound, chlorotoxin, as a novel antigen which exhibits preferential binding to GBM tumours compared to normal brain tissues, mediated by the expression of MMP-2 on tumour cells [239]. Chlorotoxin-directed CAR T-cells have demonstrated effective and specific targeting of GBM cancer cells in vivo, culminating in a Phase 1 trial (NCT05627323) [239].

However, while the development of GBM-targeting CAR T-cells have progressed on to the clinical phase, there still remains several challenges which limit the durability of patient response to these therapies. Hence, recent efforts have been directed at engineering novel CAR T-cell modalities which can overcome these issues. To overcome tumour escape and enhance tumour specificity, bivalent and trivalent CAR T-cells co-targeting two or three tumour-associated antigens have exhibited greater tumour coverage, mitigating tumour growth more efficaciously in vivo [240, 241]. Importantly, the development of IL-8 receptor-modified CD70 CAR T-cells led to greater tumour trafficking and persistence, resulting in a Phase 1 clinical trial for newly diagnosed GBM patients (NCT05353530) [242]. Interestingly, engineering bispecific CAR T-cells presenting antibodies which combine the binding domains of two antigens, such as IL-13Rα2 with either HER2, increased the antitumour effects of the T-cells [243]. Furthermore, they were shown to be superior to bivalent CAR T-cells, suggesting that these advanced bispecific CAR T-cells may hold more promise in treating GBM patients in the clinics [243]. Designing novel SynNotch-CAR T-cells which can exhibit spatially controlled activation has significantly improved the specificity and durability of CAR T-cell therapy [244, 245]. In GBM specifically, Choe et al. engineered SynNotch-CAR T-cells against EGFRvIII or myelin oligodendrocyte glycoprotein (MOG) as priming antigens, ensuring that the T cells localised to EGFRvIII- or MOG-expressing GBM tumours [244]. Subsequent expression of the IL-13Rα2/EphA2 CAR upon binding to the priming antigens induced spatially controlled and tumour-specific T-cell cytotoxicity in patient-derived in vivo models of GBM [244]. Finally, CAR T-cell therapies can be combined with immunotherapies to prevent exhaustion of T-cells, ensuring that the antitumour effects are sustained and durable. Following promising preclinical results, two clinical trials have been established to investigate the therapeutic value of co-delivering immune checkpoint inhibitors with EGFRvIII-targeting and IL-13Rα2-targeting CAR T-cells in GBM patients (NCT03726515, NCT04003649) [246]. Unfortunately however, combining EGFRvIII-targeting CAR T-cells with pembrolizumab did not confer clinical efficacy in patients, suggesting that greater efforts have to be focused on enhancing CAR T-cell therapy for GBM therapy [247].

### Oncolytic virotherapy in GBM

Oncolytic virotherapy is a strategy commonly used to target GBM tumours. Oncolytic viruses typically targets tumour cells through two complementary mechanisms. Firstly, they directly induce oncolytic lysis of the tumour cells following infection, and the new virus particles proceed to infect and lyse neighbouring target cells **(**Fig. 3C**)**. Secondly, in the process of viral infection and tumour cell lysis, several cytokines, viral pathogen-associated molecular patterns (PAMPs) and disease-associated molecular patterns (DAMPs) are released into the tumour microenvironment. This release promotes immune cell infiltration and activation, mounting an immune response against the tumours**(**Fig. 3C**)**. To date, numerous oncolytic virotherapies have been engineered from various strains of viruses and are being trialled in GBM patients, including commonly used herpes simplex virus type 1 (HSV-1) and adenoviruses, as well as polio-rhinovirus chimeras, parvoviruses, reoviruses (NCT00528684) and Newcastle disease virus [230, 248–255].

To further improve the efficacy of viral-based therapies against GBM, recent advances have been made in engineering viral strains with more complex systems. For instance, a HSV-based oncolytic virus, CAN-3110 (formerly designated rQNestin34.5v.2), was engineered to express the viral gene, *ICP34.5*, to promote viral replication and oncolysis of the tumour cells specifically by placing the gene under the transcriptional control of a nestin promoter [256–258]. This ensured that expression of *ICP34.5* was spatially confined to specifically GBM tumours which exhibit high expression of nestin and not in normal brain tissues where nestin is not expressed [259]. Given the increased specificity and enhanced potency of the virus strain, CAN-3110 is currently in a Phase 1 clinical trial (NCT03152318) [254].

Additionally, with the advent of immunotherapy in oncology, viral strains which promote immune cell trafficking and infiltration into GBM tumours have demonstrated promise in several preclinical studies. Oncolytic viruses expressing proinflammatory cytokines and antigens, such as IL-12 (HSV-1 M032) and CXCL11 (oAd-CXCL11), as well as viral particles expressing antibodies against immunosuppressive receptors including CD47 (OV-αCD47-G1) were able to elicit a strong tumour immune response and enhance the therapeutic efficacy of CAR T-cells in vivo [260–263]. Promising preclinical evidence have thus led to the assessment of HSV-1 M032, as well as IL-12 and anti-PD-1 co-expressing MVR-C5252 in clinical trials (NCT05095441) [260]. Notably, by designing viral particles with anti-EGFR cetuximab and CCL5 chimeric receptors (OV-Cmab-CCL5), Tian and colleagues were additionally able to promote the specific infiltration of various immune cells to EGFR-positive GBM tumours [264]. Collectively, these studies demonstrated the value of engineering novel viral particles to further improve the efficacy and specificity of oncolytic virotherapy in GBM.

## Combination therapies in GBM

Due to occurrence of several clonal subpopulations inside a single tumour, GBM cells frequently undergo clonal evolution in response to therapies, acquiring mutations that were not present at the time of diagnosis [265]. As a result, recurrent GBM cells frequently develop resistance to administered therapies. Furthermore, the complexity of the signalling pathway network in GBM cells also contributes to treatment resistance through a variety of processes, including acquisition of gain-of-function mutations and activation of compensatory oncogenic pathways. Therefore, combination therapeutic strategies targeting several molecular pathways serve as a potential approach to overcome drug resistance in GBM.

A common strategy for developing combination therapies in GBM involves the concurrent inhibition of the VEGF signalling pathway and a secondary oncogenic pathway triggered in response to VEGFR inhibition. Studies have reported upregulation of the TGF-β–CD105–Smad pathway as an alternative angiogenic pathway that contributes to BEV resistance in GBM [266–268]. This suggests that targeting the ligand, endoglin (CD105), may be a potential therapeutic approach. However, neither clinical trials evaluating the combination of BEV and anti-endoglin agent, TRC105, received positive responses in patients [269, 270]. Concurrent inhibition of VEGFR and the PI3K/AKT/mTOR pathway is an alternative strategy as previous studies have shown that the aberrant activation of PI3K/AKT/mTOR signalling stimulates excessive secretion of VEGF [271, 272]. However, such combinations did not exhibit promising clinical value for GBM patients. In a Phase 1/2 study of BEV with PI3K inhibitor, buparlisib, the combination was poorly tolerated at low doses of buparlisib, and it failed the trial with unsatisfactory PFS of only 4 months and an overall response rate (ORR) of 26% [273]. Similarly, simultaneous inhibition of VEGFR and mTOR via BEV and everolimus did not yield promising results, with no significant improvement in patients’ PFS and OS [274]. Ongoing trials are thus underway to evaluate the efficacy of BEV in combination with new generation mTOR inhibitors such as nab-sirolimusand sapanisertib, which are predicted to yield more promising results [275, 276]. More clinical trials are ongoing to evaluate the efficacy of BEV with other potential therapeutic agents including the emerging PD-1 inhibitors, angiopoietin1/2 inhibitors, proteasome inhibitors, and multi-kinase inhibitors (Table 2).

The PI3K/AKT/mTOR is another crucial signalling pathway that is often hyperactive and complexed with multiple feedback loops. In one study, MK-2206 suppression of p-AKT was observed to cause aberrant activation of mTOR and radiation resistance in PTEN-deficient GBM cells, implying that the efficacy of AKT inhibitors may be limited by the negative feedback loop that increases mTORC1 activity in GBM cells [277]. This suggests that dual-inhibition of AKT and mTOR may be a more effective approach in targeting GBM cells. This has led to ongoing clinical trials investigating the efficacy of perifosine in combination with temsirolimus in recurrent malignant gliomas following patient tolerance in a Phase 1 trial [278] (Table 2). Combination therapies with mTOR inhibitors and other drugs are also undergoing investigations. The combination of everolimus and CDK4/6 inhibitor, ribociclib, was tested in two clinical trials, both suggesting that the drug combination was well-tolerated and may achieve therapeutic effects [279, 280]. Multiple trials have also looked into the potency of mTOR inhibitors in combination with multi-kinases inhibitors, although initial assessment with first generation kinase inhibitors did not yield clinical benefit for patients. Everolimus in combination with gefitinib has failed a Phase 1/2 trial with unsatisfactory antitumour activity [281], while the combination of temsirolimus and sorafenib received considerable grade 3 + toxicities in a Phase 1/2 trial [282]. On the contrary, a Phase 1 trial which evaluated the combination of sirolimus and vandetanib has demonstrated that the two drugs can be co-administered safely, suggesting that only specific pairs of mTOR inhibitors and multi-kinase inhibitors would present satisfactory safety profiles in patients [283].

The therapeutic effects of proteasome inhibitors have been investigated in many clinical trials as a single agent. Based on strong preclinical evidence of disulfiram as a therapeutic agent in combination with TMZ [284–286], the safety profile of disulfiram in combination with Stupp protocol and copper was investigated in two clinical trials [184, 287] (Table 1). In contrary to preclinical results, though the drug combination was found to be well-tolerated in both newly diagnosed and recurrent GBM patients, it conferred minimal proteasome inhibition, and no overall benefits over the control group [183, 287]. The efficacy of this drug combination is being further investigated in two ongoing trials [184, 288] (Table 1). Previous preclinical study has also demonstrated that inhibition of proteasomes could induce HIF1α and VEGF production in malignant GSCs, thus suggesting that co-inhibition of the proteasome and VEGF could achieve better tumour inhibition [289]. However, the efficacy of BEV in combination with either bortezomib or marizomib did not yield meaningful benefits in two independent trials (NCT00611325) [176].

Despite much effort to develop combination therapies for GBM patients, there is still no FDA-approved combination targeted therapy for GBM patients, with few generating promising results. There is thus a need to develop and identify novel combinations which may have therapeutic potential in GBM. In addition to the complex network of feedback mechanisms that drive drug resistance and compensatory pathways in GBM, the incomplete knowledge in underlying molecular interactions makes it challenging to identify novel optimal drug combinations from a pool of potential drug candidates. To overcome the challenges in conventional drug combination design, various models and algorithms have been developed to predict potential synergistic drug combinations using smaller datasets. One such platform is the quadratic phenotypic optimization platform (QPOP) which identifies the best drug combination for each patient via second-order linear regression analysis without prior knowledge of the molecular mechanism of the drugs [290]. Other computational techniques designed to predict drug synergism include the Feedback System Control (FSC) [291], Markov chain-based models [292] and the drug combination network (DCN) [293]. Interestingly, recent efforts to identify novel drug combinations in GBM have been successful in identifying novel drug combinations and repurposing FDA-approved drugs for the inhibition of GBM. The computational platform SynergySeq integrates transcriptional data with perturbagen-induced transcriptional signatures to identify the novel synergistic combination of BRD4 inhibitor, JQ1, and aurora kinase inhibitor, alisertib, in mitigating GBM growth in vivo [294]. The study additionally identified the combination of FDA-approved gemcitabine and imatinib for the treatment of GBM, offering novel combination therapies for GBM, which may otherwise not be investigated [294]. While such combinations require further validation and clinical assessment, this study supports the use of computational tools to identify promising combination therapies for GBM.

Although combination therapy is a promising approach to treat GBM, there are many challenges in finding and testing novel drug combinations. Apart from the potential toxicity that arises from the use of several drugs, many clinical trials may have failed due to the lack of patient stratification. As tumour heterogeneity is one of the most important hallmarks of GBM, it is expected that GBM patients display varied drug sensitivity, and their responses to the same targeted treatment is bound to be diverse. It is therefore imperative to adopt selection strategies such as molecular-based selection or pathway-based stratification in clinical trials to identify specific patient cohorts who can attain clinical benefit from the respective targeted therapies [295, 296]. Furthermore, with advancements in imaging techniques, imaging-based biomarkers can potentially be developed to investigate target engagement and treatment response as a strategy to stratify patient sensitivity [297–300].

## Conclusion

In conclusion, the identification of key genetic mutations and their roles in oncogenesis in GBM has paved the way for robust research and drug discoveries in the field of targeted therapies and has presented a positive outlook in improving clinical benefit for GBM patients. However much still has to be done to significantly improve patient response. Given the heterogeneous nature of GBM, a future challenge is the prioritization of target combinations to overcome therapy resistance arising from cross-talks between various signalling pathways. Additionally, endeavours to stratify patients according to their molecular characteristics will greatly improve the identification of patient cohorts who exhibit greater sensitivity to corresponding targeted therapies and combinations. Future efforts to develop therapeutic strategies concurrent with the incorporation of specific molecular and imaging biomarkers will significantly improve the treatment outcome of GBM patients in the clinics.

## Data Availability

No datasets were generated or analysed during the current study.

## Abbreviations

4EBP1: Eukaryotic translation initiation factor 4E-binding protein 1
5-FC: 5-fluorocytosine
5-FU: 5-fluorouracil
5-hmc: 5-hydroxymethylcytosine
5-mc: 5-methylcytosine
AMPK⍺: AMP-activated protein kinase ⍺
ASO: Antisense oligonucleotides
ATM: Ataxia-telangiectasia mutated
BAX: Bcl-2-associated X
BBB: Blood–brain barrier
Bcl-2: B-cell lymphoma 2
Bcl-XL: B-cell lymphoma-extra large
BCL2: B-cell lymphoma 2
Bcl-XL: B-cell lymphoma-extra large
BCL2: B-cell lymphoma 2
BER: Base excision repair
BFABP: Brain fatty acid-binding protein
BRCA: BReast CAncer gene
CD: Cytosine deaminase
CNS: Central Nervous System
CP: Core particle
D-2-HG: D-2-hydroxyglutarate
DAG: Diacylglycerol
DBS: Double-strand break
DMR: Differentially methylated region
DSB: Double strand break
EGFR: Epidermal Growth Factor Receptors
elF4F: Eukaryotic initiation factor 4 F
EMT: Epithelial-mesenchymal transition
EMT: Epithelial-mesenchymal transition
ERBB2: Erb-B2 receptor tyrosine kinase 2
ERK: Extracellular signal-regulated kinase
FAK: Focal adhesion kinase
FSC: Feedback system control
G-CIMP: Glioma CpG island methylator phenotype
GBM: Glioblastoma
GQ-ODN: G-Quartet Oligonucleotides
GSC: Glioblastoma stem cells
H4K9: :Histone 3 lysine 9
HIF-1: Hypoxia-Inducible Factor 1
HR: Homologous recombination
IDH: Isocitrate dehydrogenase
JAK: Janus tyrosine kinase
KDM4: Histone lysine demethylase 4
KDM5: Histone lysine demethylase 5
MAPK: Mitogen-activated protein kinase
Mcl-1: Myeloid leukaemia 1
MGMT: O^6^-alkylguanine DNA alkyltransferase
MMK: Mitogen-activated protein kinase (MAPK) kinase
MMP: Matrix metalloproteinases
MMR: Mismatch repair
MSC: Mesenchymal stem cell
mTOR: Mammalian target of rapamycin
NF1: Neurofibromin 1
NSC: Neural stem cell
OS: Overall survival
PARP: Poly(ADP-ribose) polymerase
PD-1: Programmed cell death protein 1
PDGFR: Platelet-derived growth factors
PFS: Progression-free survival
PI3K: Phosphoinositide 3-kinase
PI3KCA: Phosphatidylinositol-4,5-Bisphosphate 3-Kinase Catalytic Subunit Alpha
PIK3R1: Phosphoinositide-3-kinase regulatory subunit 1
PKC: Protein kinase C
PTEN: Phosphatase and tensin homolog deleted on chromosome ten
QPOP: Quadratic phenotypic optimization platform
RTK: Receptor tyrosine kinases
S6k: Ribosomal protein S6 kinase
SH2: Src homology 2
STAT3: Signal transducer and activator of transcription 3
TERT: Telomerase reverse transcriptase
TET: Ten-eleven translocation
TGF-β: Transforming growth factor beta
TMZ: Temozolomide
TP53: Tumour protein P53
TRAIL: TNF-related apoptosis-inducing ligand
ULK1: Unc-51-like kinase 1
UPP: Ubiquitination-proteasome pathway
VEGFR: Vascular endothelial growth factor receptor
